# Aerobic granular sludge for complex heavy metal-containing wastewater treatment: characterization, performance, and mechanisms analysis

**DOI:** 10.3389/fmicb.2024.1356386

**Published:** 2024-01-31

**Authors:** Chong Liu, Yao Shen, Yuguang Li, Fengguang Huang, Shuo Wang, Ji Li

**Affiliations:** ^1^Key Laboratory of Embalming Methodology and Cosmetology of Cadavers of the Ministry of Civil Affairs, 101 Institute of the Ministry of Civil Affairs, Beijing, China; ^2^Jiangsu Key Laboratory of Anaerobic Biotechnology, School of Environment and Ecology, Jiangnan University, Wuxi, China; ^3^Jiangsu College of Water Treatment Technology and Material Collaborative Innovation Center, Suzhou, China

**Keywords:** complex heavy metals, cyclic diguanylate, extracellular polymeric substances fractions, phosphodiesterases, removal mechanisms, aerobic granular sludge

## Abstract

Complex heavy metal (HM)-containing wastewater discharges pose substantial risks to global water ecosystems and human health. Aerobic granular sludge (AGS) has attracted increased attention as an efficient and low-cost adsorbent in HM-containing wastewater treatment. Therefore, this study systematically evaluates the effect of Cu(II), Ni(II), and Cr(III) addition on the characteristics, performance and mechanism of AGS in complex HM-containing wastewater treatment process by means of fourier transform infrared spectroscopy, inductively coupled plasma spectrocopcy, confocal laser scanning microscopy, extracellular polymeric substances (EPS) fractions detection and scanning electron microscope-energy dispersive X-ray. The results showed that AGS efficiently eliminated Cu(II), Ni(II), and Cr(III) by the orchestrated mechanisms of ion exchange, three-layer EPS adsorption [soluble microbial products EPS (SMP-EPS), loosely bound EPS (LB-EPS), tightly bound EPS (TB-EPS)], and inner-sphere adsorption; notably, almost 100% of Ni(II) was removed. Three-layer EPS adsorption was the dominant mechanism through which the HM were removed, followed by ion exchange and inner-sphere adsorption. SMP-EPS and TB-EPS were identified as the key EPS fractions for adsorbing Cr(III) and Cu(II), respectively, while Ni(II) was adsorbed evenly on SMP-EPS, TB-EPS, and LB-EPS. Moreover, the rates at which the complex HM penetrated into the granule interior and their affinity for EPS followed the order Cu(II) > Ni(II) > Cr(III). Ultimately, addition of complex HM stimulated microorganisms to excrete massive phosphodiesterases (PDEs), leading to a pronounced decrease in cyclic diguanylate (c-di-GMP) levels, which subsequently suppressed EPS secretion due to the direct linkage between c-di-GMP and EPS. This study unveils the adaptability and removal mechanism of AGS in the treatment of complex HM-containing wastewater, which is expected to provide novel insights for addressing the challenges posed by intricate real wastewater scenarios.

## 1 Introduction

Accelerating urbanization and industrialization have resulted in large quantities of heavy metals (HM) wastewater with high toxicity, bio-accumulation, and non- biodegradability being discharged into municipal wastewater treatment plants (WWTPs), which poses a formidable challenge to the wastewater treatment industry (Cantinho et al., 2016; Anderson et al., 2022; Wu D. et al., 2023; Wu X. et al., 2023). Cu(II), Ni(II), and Cr(III) are common HM ions in wastewater of the electroplating, smelting, alloy manufacturing, and battery industries, which can seriously damage human health worldwide (Hu et al., 2019; Thomas et al., 2021). For example, Cu(II) toxicity leads to liver and kidney damage, while Ni(II) and Cr(III) are both carcinogens that cause lung cancer and tumors, among other diseases (Tchounwou et al., 2012; Fei and Hu, 2023). In response, the World Health Organization (WHO) has set the Ni(II) and Cr(III) thresholds in drinking water to 0.1 mg/L and 100 ug/L, respectively (Sun et al., 2014; World Health Organization, 2020). Therefore, it is critical to eliminate toxic HM from wastewater based on environmental and health considerations.

Accordingly, various technologies have been developed to remove HM from the ecosystem, including chemical precipitation, ion exchange, membrane technology, or solvent extraction. However, these conventional methods have limitations regarding low efficiency, high operating costs, sensitive operating status, and toxic sludge production (Foroutan et al., 2021; Shahrokhi-Shahraki et al., 2021). In contrast, biosorption has emerged as a promising alternative for treating HM-containing wastewater on account of its easy implementation, efficiency, and cost-effectiveness (Anderson et al., 2022). In particular, aerobic granular sludge (AGS) has attracted increased attention as an efficient and low-cost adsorbent in HM-containing wastewater treatment (Wang et al., 2018). A comparison of AGS and anaerobic granular sludge revealed that the former consistently exhibited higher biosorption levels of HM Ni(II) than the latter (Li et al., 2017). Moreover, the maximum adsorption capacity of AGS for Cr(IV) (348.125 mg/g) was remarkably higher than that of Cu(II) (246.1 mg/g) (Purba et al., 2020). To date, the majority of studies have validated the applicability of AGS for efficient removal of single HM such as Cu(II), Ni(II), Cr(III), Zn(II), and Pb(II) (Wang et al., 2018; Purba et al., 2020). However, the mechanisms of AGS in treating complex HM-containing wastewater have not been fully elucidated.

Moreover, extracellular polymeric substances (EPS) play an instrumental role in the removal of HM from wastewater due to the presence of numerous adsorption sites and functional anionic groups (e.g., carboxyl, hydroxyl, amino groups) located on the EPS surface (Li and Yu, 2014; Hasani Zadeh et al., 2023). EPS consist of components such as proteins (PN), polysaccharides (PS), nucleic acids, lipids, and humic substances, accounting for up to 90% (w/w) of the organics in biofilms (Flemming et al., 2016). EPS are categorized into soluble microbial products EPS (SMP-EPS), loosely bound EPS (LB-EPS), and tightly bound EPS (TB-EPS) with respect to their association with the cell surface, forming an ordered protective shield around microbial cells joint against environmental stressors, including toxic HM (Nouha et al., 2018; Hasani Zadeh et al., 2023). Previous studies have indicated that PN exhibited a markedly higher adsorption capacity for Cu(II), Zn(II), and Cd(II) than PS (Wei et al., 2019). Further metrological evidence suggests that the carboxyl groups of PN have the fastest response in binding Cu(II) in comparison to PS and hydrocarbons (Li G. et al., 2020; Li N. et al., 2020). The importance of EPS was also demonstrated by their efficient removal of various HM ions such as Cu(II) (90%), Cr(VI) (88%), Ni(II) (65%), and Pb(II) (73%) from simulated electroplating and synthetic wastewater (Siddharth et al., 2021). Of note, cyclic diguanylate (c-di-GMP), an intracellular messenger, plays a critical role in regulating EPS production and maintaining granular stability (Wan et al., 2013; Liang, 2015), but little has been reported on its relevance to EPS and the effects of HM stress. In summary, while research on EPS adsorption and HM removal has made significant progress, the contribution of different EPS fractions and the transformation mechanism remain understudied. Furthermore, the detailed mechanism by which AGS removes complex HM needs further clarification.

Therefore, this study systematically looked into the effect of Cu(II), Ni(II), and Cr(III) addition on the characteristics and performance of AGS treatment of complex HM-containing wastewater by means of fourier transform infrared (FTIR) spectroscopy, inductively coupled plasma (ICP) spectroscopy, confocal laser scanning microscopy (CLSM), and scanning electron microscope-energy dispersive X-ray (SEM–EDX). Furthermore, the removal mechanism of complex HM by AGS and the transformation mechanism in SMP-EPS, LB-EPS, and TB-EPS were elucidated. This study advances our knowledge on AGS for complex HM-containing wastewater treatment toward practical applications.

## 2 Materials and methods

### 2.1 Synthetic wastewater

Synthetic wastewater with the following composition was used in this study: 8,560 mg/L NaAc, 2,240 mg/L NH_4_Cl, 288 mg/L KH_2_PO4, 735 mg/L K_2_HPO_4_, 1804 mg/L MgSO_4_·7H_2_O, 350 mg/L KCl, and 1.0 mL/L trace element solution. The trace element solution contained 40 g/L EDTA, 150 mg/L H_3_BO_3_, 120 mg/L MnCl_2_·4H_2_O, 30 mg/LCuSO_4_·5H_2_O, 120 mg/L ZnSO_4_·7H_2_O, 60 mg/L Na_2_MoO_4_·2H_2_O,1,500 mg/L FeSO_4_·7H_2_O,and 150 mg/LCoCl_2_·6H_2_O. The initial concentrations of COD, NH_4_^+^-N, and PO_4_^3−^-P were 600 mg/L, 60 mg/L, and 16 mg/L, respectively. When the mature AGS systems had been acclimatized to the feed, CuCl_2_·2H_2_O, NiCl_2_·5H_2_O, and K_2_Cr_2_O_4_ solutions were added to the synthetic wastewater from a carbon source tank to provide 50 mg/L Cu(II), 5 mg/L Ni(II), and 3 mg/L Cr(III) to the reactor.

### 2.2 Reactor set-up and operation

Sludge seeded in this study was taken from the aerobic tank of the Lucun municipal wastewater plant in Wuxi City, which treats wastewater using an anaerobic-anoxic-oxic (A^2^O) process. The sludge was cultivated in a 4-L sequencing batch reactor (SBR, 100 cm in height and 10 cm in diameter) at room temperature, and sampling points were arranged at the top, middle, and bottom of the reactor (Supplementary Figure S1). In order to avoid the microbial growth of each component affecting the influent quality after water distribution, the SBR reactor unit uses three tanks of carbon source, nitrogen and phosphorus source and pure water in the ratio of 2:2:6 to be input from the bottom of the reactor. The reactor was fed from the bottom by a peristaltic pump, and the effluent was discharged from the middle part of the reactor with an aeration rate of 2 L/min and a volumetric exchange ratio of 50%. The initial suspended solid (SS), sludge volume index (SVI), and solids retention time (SRT) were 2.5 g/L, 110 mL/g, and 21 d, respectively. The operating cycle of 4 h consisted of 60 min of feeding, 170 min of aeration, variable settling time (from 30 min to 5 min) and effluent drainage.

### 2.3 Analytical methods

#### 2.3.1 EPS fractions determination

The three main components of EPS were extracted by a formamide-NaOH method (Liang et al., 2010), comprising SMP-EPS, LB-EPS, and TB-EPS. The extraction procedure of each EPS fraction is as follows: dissolve a certain amount of sludge in 20 mL of distilled water, sonicate at 40 W for 1 min; take out the sludge and centrifuge at 4°C, 2,000 g for 15 min, and then pass the supernatant through a 0.22 μm membrane to obtain the SMP-EPS. The centrifuged substrate was mixed with 10 mL of distilled water, added with 5 mL of 1 mol/L NaOH solution, and shaken in a shaker at 4°C for 3 h. The resulting mixture was centrifuged at 4°C, 10,000 g for 15 min, and the supernatant was passed through a 0.22 μm filter membrane, and TB-EPS was obtained. The supernatant was filtered through 0.22 μm filter membrane, and then TB-EPS was obtained. The PS and PN content were qualified with the anthrone-sulfuric acid and coomassie brilliant blue methods, respectively (Adav and Lee, 2008; Wang et al., 2017a).

#### 2.3.2 CLSM observation

CLSM observes the changes of complex components of sludge by multiple fluorescence labeling method (Wang et al., 2016). In this study, the Syto63, Sytox blue, fluorescein isothiocyanate (FITC), Nile red, Concanavalin A (ConA), and Calcofluor white (CW) were used to label the total cells, dead cells, proteins, lipids, α-polysaccharides, and β-polysaccharides in sludge, respectively.

The staining procedure for each component was as follows: 40 μL of 20 mol/L Syto63 was added to the centrifuge tube with the sludge samples, shaken for 30 min, and washed twice with deionized water; 200 μL of 1 mol/L NaHCO_3_ solution at PH = 9 was added to the centrifuge tube, 20 μL of 10 g/L FITC stain was added, and the tube was shaken for 1 h. Then add 100 μL of 0.25 g/L ConA reagent, shake for 30 min; then add 100 μL of 300 mg/L CW dye, shake for 30 min. Subsequently, add 60 μL of 10 mg/L Nile Red dye, shake for 10 min. Finally, 60 μL of 25 mol/L Sytox Blue dye was added, shaken for 5 min. After the above six staining stages, the samples were washed twice with phosphate buffered saline (PBS) to remove excess stain. The stained samples were cryosectioned at −50°C and meso-sections with a thickness of 60 μm were placed under a microscope for observation. CLSM (Leica TCS SP8, Gmbh, Germany) was used to probe the internal structure and components of the granules. The total cells, dead cells, proteins, lipids, α-polysaccharides, and β-polysaccharides were probed by excitation/emission wavelengths of 633 nm/650–700 nm, 458 nm/460–500 nm, 488 nm/500–540 nm, 514 nm/625–700 nm, 543 nm/550–600 nm and 400 nm/410 nm-480 nm, respectively.

#### 2.3.3 PDEs activity detection

The activity of PDEs was determined by p-nitrophenyl phosphate method. The procedure was as follows: 1 g of filtered sludge was weighed into a 50 mL conical flask, and 0.2 mL of toluene (C_7_H_8_), 4 mL of tris (hydroxymethyl) aminomethane buffer (THAM buffer, pH = 8.0) and 1 mL of sodium p-nitrophenyl phosphate were added to the sludge and incubated in an incubator at 37°C for 1 h. The sludge was then taken out of the incubator, and then 1 mL of CaCl_2_ with the concentration of 0.5 mol/L was added to it, and 4 mL of THAM-NaOH leachant (THAM-NaOH). The solution was filtered through 0.45 μm filter paper and colorimetrically measured at 410 nm. The content of p-nitrophenol in the solution was calculated from the standard curve, and then the yield of p-nitrophenol per unit time was calculated according to the following formula, which was used to evaluate the PDEs activity.

$$\omega \left(C_{6}H_{5}NO_{3}\right)=\frac{m_{1}}{k\times m_{2}}$$

where *ω* is the yield of p-nitrophenol per unit time (mg/g-h); *m*_1_ is the mass of p-nitrophenol produced in the sample (mg); *m*_2_ is the mass of the sample to be tested (g) and *k* is the water content of the sample to be tested.

#### 2.3.4 Other methods

Chemical oxygen demand (COD), ammonium-N (NH_4_^+^-N), phosphorus (PO_4_^3−^-P), nitrite-nitrogen (NO_2_^−^-N), nitrate-nitrogen (NO_3_^−^-N), SS, volatile suspended solids (VSS), and SVI were measured as standard methods (APHA, 2005). Cu(II), Cr(III), Ni(II), Ca(II), Mg(II), and K(I) concentrations in solution were analyzed by ICP emission spectrometry. The distribution of adsorbed HM in aerobic granules was determined using SEM–EDX. FTIR spectra before and after metal adsorption tests were recorded using a FTIR spectrometer (Avatar 370, USA). The c-di-GMP content was extracted as described before (Wan et al., 2013). The micro-structure was visualized using SEM (SEM, JEOLJSM 5310).

## 3 Results and discussion

### 3.1 Effect of complex HM on AGS morphology

#### 3.1.1 Macroscopic morphology and characteristics of granules

A gradual color change from yellow to pale greenish was observed for granules on the 3rd day following Cu(II), Ni(II), and Cr(III) addition (Supplementary Figures S2A,B), which was likely due to the fact that certain HM were adsorbed on the granule surface. Meanwhile, a minor portion of the disintegrated granules was discharged, resulting in a decrease in SS from 8 to 5 g/L (Supplementary Figure S3A). The reduction may also be related to the inhibitory effect of high concentrations of Cu(II) (50 mg/L), as 5 mg/L of Cu(II) was sufficient to inhibit biomass growth (Wang et al., 2010). As the reactor continued to operate, a black substance appeared in the granule interiors on the 10th day (Supplementary Figure S2C), which was speculated to be precipitates generated by the reaction of HM entering the granule interiors through pore channels. Long-term operation resulted in these precipitate being selectively discharged (Supplementary Figure S2D), which preserved the excellent settling performance in the SBR system (Supplementary Figure S3A). In comparison to the initial granules, those augmented with complex HM exhibited a significant increase in size and maintained structural integrity. This phenomenon could possibly be ascribed to absorption of HM by AGS through ion exchange, leading to the release of light metals such as Ca(II) and Mg(II) to maintain the granular compact morphology (Kończak et al., 2014). Overall, the addition of complex HM appeared to have a negligible impact on the settling performance of the granules but significantly altered their apparent morphology.

#### 3.1.2 Micro-structure and compositional analysis of granules

**Figure 1:**
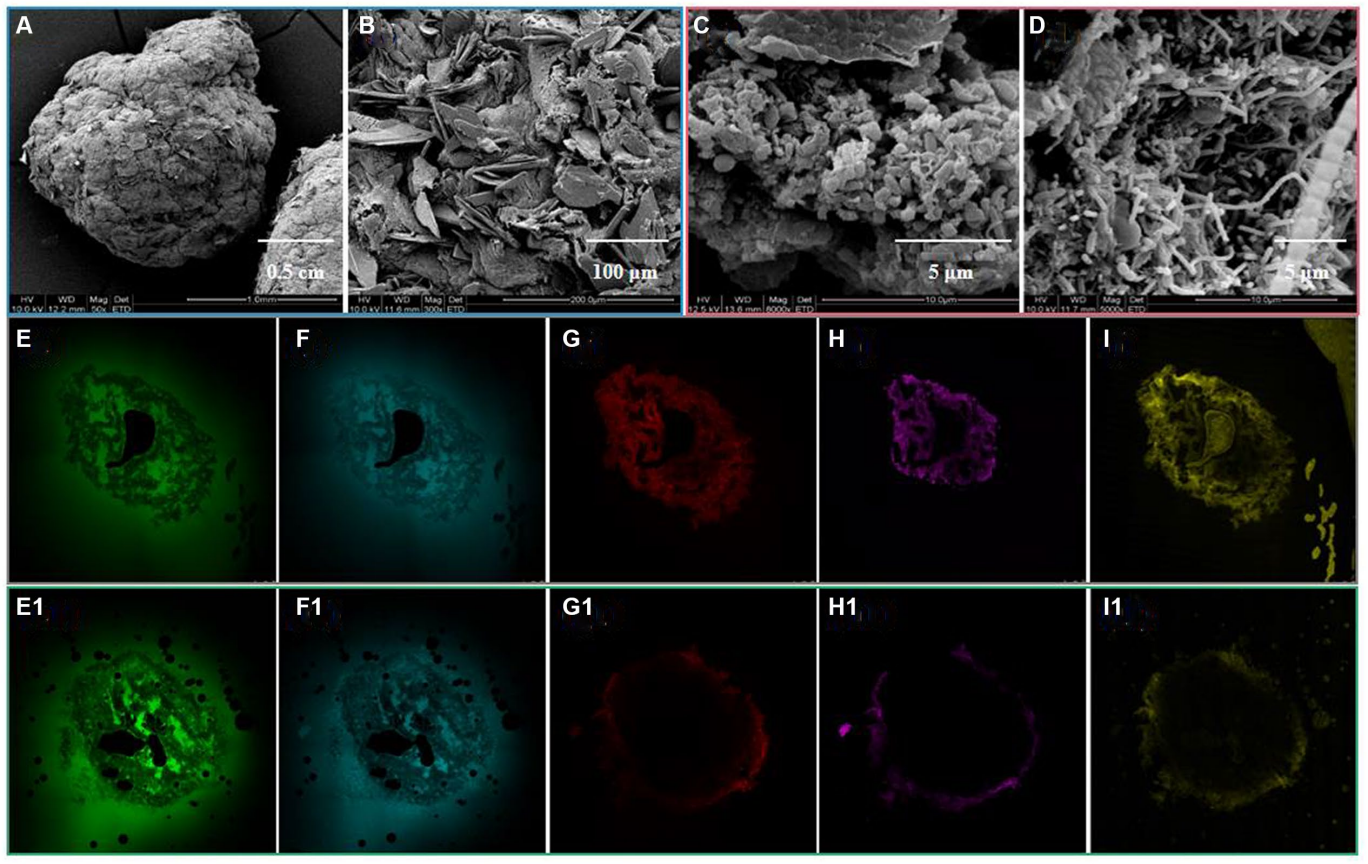
Micro-structure of granule surface **(A,B)** and interior **(C,D)** after HM addition, and the distribution of PN **(E,E1)**, PS **(F,F1)**, lipids **(G,G1)**, total cells **(H,H1)**, and dead cells **(I,I1)** of granular sludge before and after HM addition.

**Figure 2:**
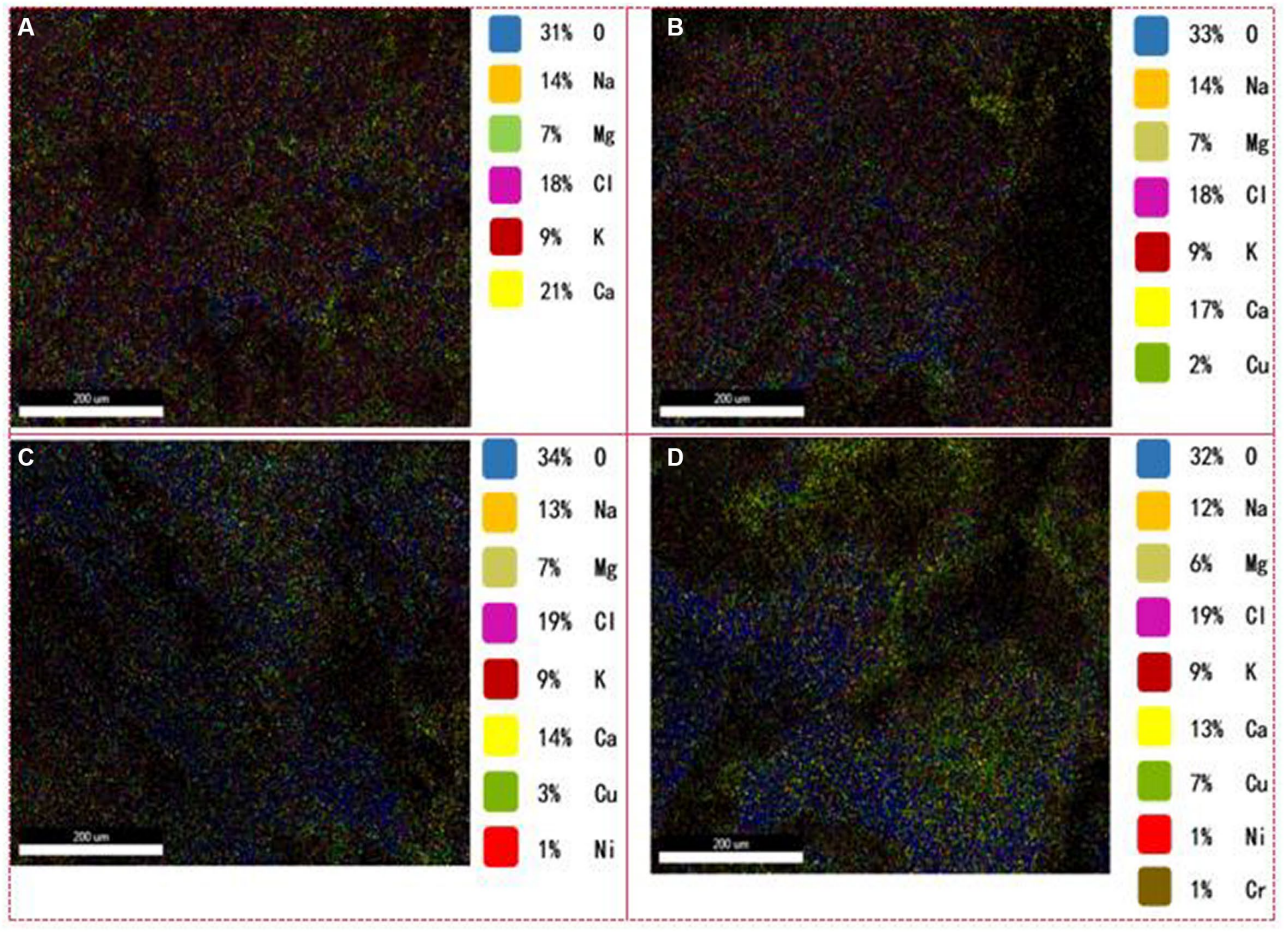
Distribution of metal ions in the granule interiors. **(A)** Without HM addition; **(B)** HM addition for 1 week; **(C)** HM addition for 2 weeks; **(D)** HM addition for 3 weeks.

As depicted in Figure 1A, the introduced complex HM did not disrupt the stable spherical structure of the aerobic granules. Notably, the porous surface structure of the granules vanished, as seen when compared to their initial state, giving way to a denser composition, which was likely a consequence of the absorption of HM in the initial pore spaces. A plethora of scale-like substances were also observed on the granule surface (Figure 1B), presumably caused by HM adsorption and precipitation. A few similar scale-like entities were detected in the granule interiors (Figure 1C), signifying diffusion of HM through the pores and their subsequent absorption into the granule interior (Ahn and Hong, 2015), as corroborated by the SEM–EDX results in Section 3.4.4 (Figure 2). Additionally, an elevated concentration of Cu(II) appeared to stimulate the growth of filamentous bacteria, giving rise to loosen the internal granular structure, as displayed in Figure 1D (Purba et al., 2020).

An in-depth observation by means of CLSM of the distribution of PN, PS, lipids, total cells, and dead cells of granular sludge before and after HM addition revealed that HM primarily poisoned microbial cells, slightly sparing PN and PS (Figure 1), which maintained the stable structure of the granules under HM stress. Following the addition of HM, an overlap between total and dead cells on the granular outside occurred (Figures 1H,H1,I,I1), indicating diminished microbial activity owing to the accumulation of HM toxicity. This reduced microbial activity further led to decreased lipid secretion, as shown in Figures 1G,G1.

### 3.2 Reactor performance under complex HM stress

#### 3.2.1 Pollutant removal performance

**Figure 3:**
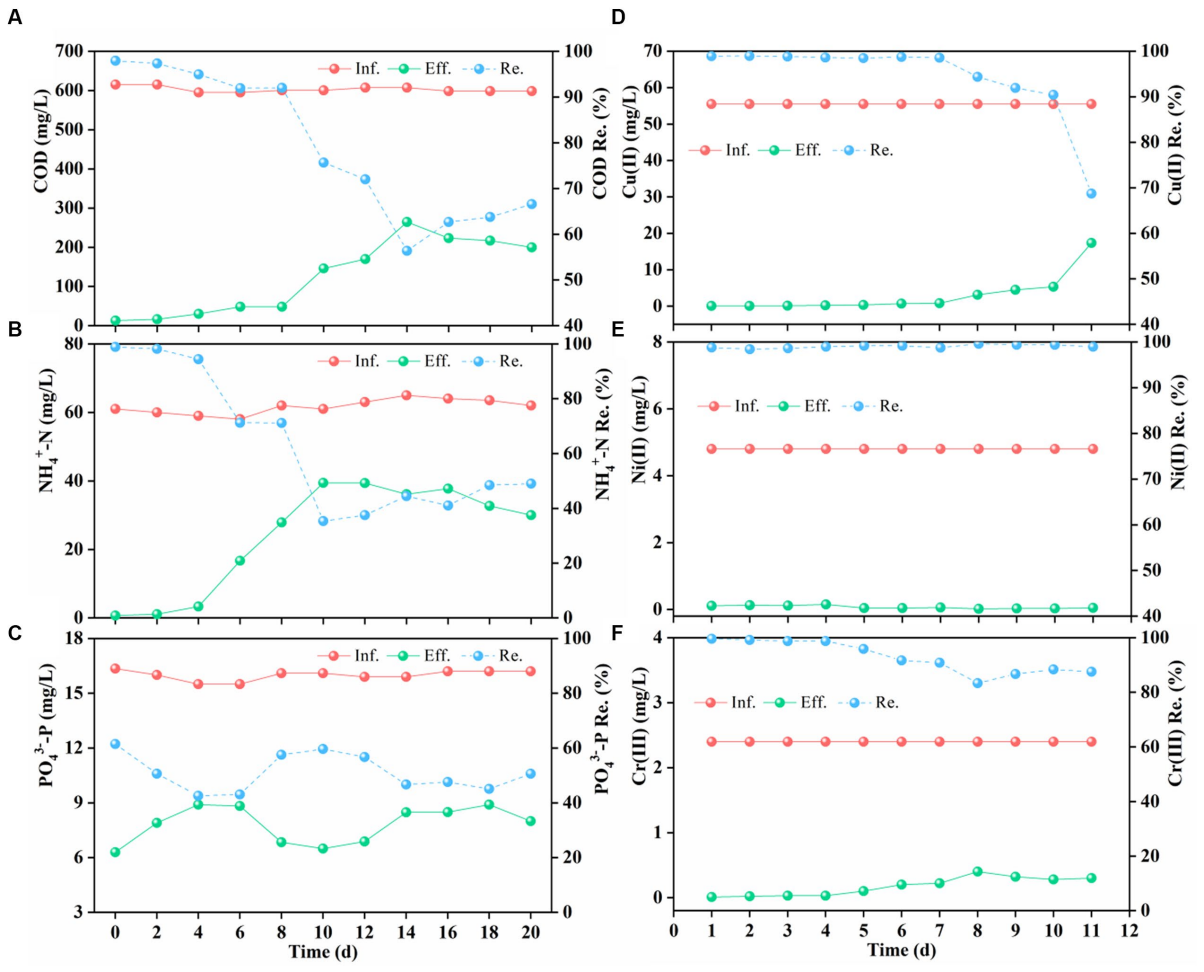
Inffuent (Inf.) and efffuent (Eff.) concentration, removal efffciency (Re.) of chemical oxygen demand (COD) **(A)**, ammonium-N (NH_4_^+^-N) **(B)**, phosphorus (PO_4_^3−^-P) **(C)**, Cu(II) **(D)**, Ni(II) **(E)**, and Cr(III) **(F)** after HM addition.

Figure 3 reveals that addition of complex HM significantly inhibited pollutant removal, while AGS exhibited adaptive and rebound trends in later stages. As shown in Figure 3A, AGS efficiently removed COD (>90%) during the initial phase of HM addition, but the efficiency dropped to 60% given rise to the impact of HM accumulation after one week, which corroborates previous findings that Cu(II) inhibits COD degradation (Su et al., 2022). Following gradual adaptation, COD removal rebounded and stabilized around 70%. Analogously, NH_4_^+^-N removal was initially at 95% but plummeted to below 40% after one week (Figure 3B), indicating suppression of ammonia-oxidizing bacteria by HM. Strikingly, effluent NO_3_^−^-N concentrations sharply rose initially, while those of NO_2_^−^-N remained stable at a lower level (Supplementary Figure S3B), possibly due to inhibition of denitrification by Cr(III) and Ni(II) (Ramírez et al., 2018; Jiang et al., 2020). Evidently, compared to nitrifying bacteria, denitrifying bacteria proved more sensitive to HM stress. Additionally, PO_4_^3−^-P removal rates experienced a slight decline under HM stress (Figure 3C), which was mainly attributed to the suppression of phosphorus-accumulating organisms (PAO) by complex HM.

#### 3.2.2 HM removal performance

The addition of Cu(II), Ni(II), and Cr(III) not only affects organic and nutrient removal but also poses a significant threat to human health as their non-biodegradability. Figure 3 illustrates the performance of AGS in removing Cu(II), Ni(II), and Cr(III). As shown in Figure 3D, Cu(II) was removed almost entirely within the first week, but the removal efficiency rapidly declined to around 70% owing to its accumulation at toxic levels. In contrast, the removal efficiency of Ni(II) was maintained consistently high at nearly 100% (Figure 3E), indicating its stimulating effect on aerobic granular activity (Ong et al., 2004). Previous studies have also discovered that Ni(II) exerted no significant toxic effects in the AGS system, even at a high-concentration of 15 mg/L (Wang et al., 2010). In addition, AGS efficiently removed Cr(III) (>90%) in the initial stages (Figure 3F), reducing the effluent content to as low as 0.1 mg/L, which meets the discharge standards in the EU and USA (Vaiopoulou and Gikas, 2020). Although Cr(III) removal mildly declined after 4 d, it rebounded to approximately 90% after adaptation, showcasing long-term efficiency of AGS in Cr(III) removal. Despite the high-concentrations of complex HM in the wastewater, AGS had varying removal efficiencies for Cu(II), Cr(III), and Ni(II). Ni(II) was almost entirely removed, while Cr(III) and Cu(II) removal rates decreased after the 4th and 7th day, respectively, with Cu(II) showing a more significant decrease. This indicated that Cr(III) disrupted AGS more rapidly, whereas Cu(II) demonstrated higher toxicity. Overall, AGS presented superior removal performance and adaptability for complex HM compared to conventional activated sludge (Yang et al., 2015).

### 3.3 EPS and c-di-GMP in response to complex HM stress

#### 3.3.1 Variations in EPS content and fractions

**Figure 4:**
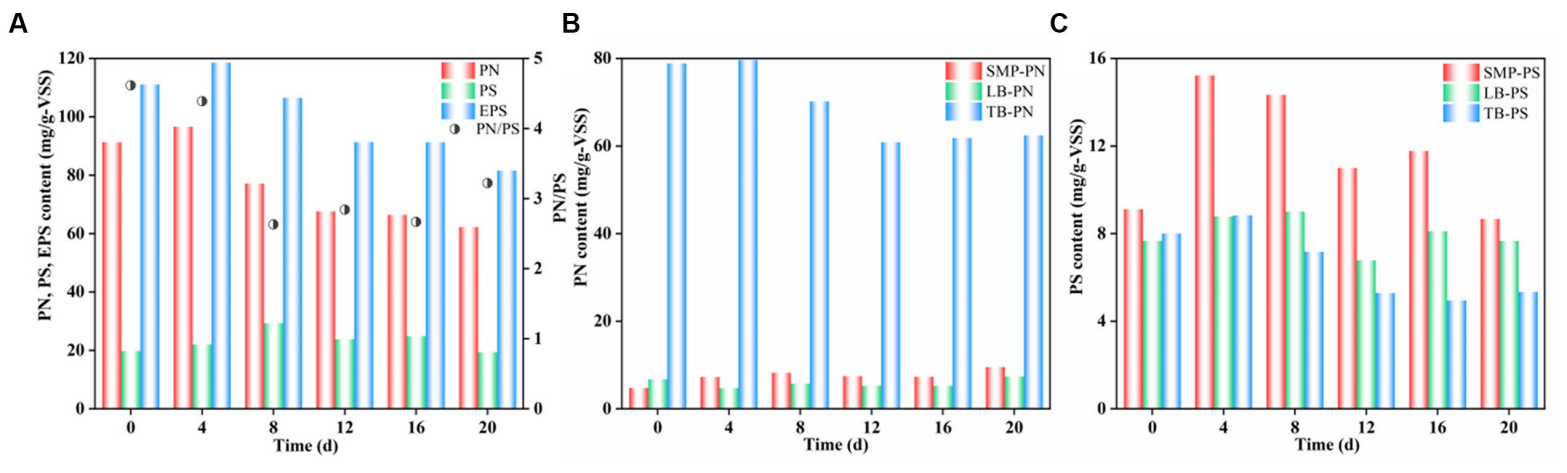
Variation in concentrations of EPS fractions after HM addition.

EPS play a vital role in AGS formation, being tightly linked to microbial aggregation and biosorption (Li and Yu, 2014; Wan et al., 2022). Figure 4 presents the impact of HM addition on the EPS content and fractions per layer. An increase in EPS contents during the initial period of HM addition is a natural response of microorganisms exposed to toxic environments, serving as a protective mechanism for cells to cope with adverse external conditions (Sheng et al., 2005). As the reactor operation, the accumulation of HM might have exceeded the capacity of EPS to bind metal cations, leading to a weaker microbial activity and a subsequent decrease in EPS contents, which eventually stabilized at approximately 80 mg/g-VSS (Figure 4A). Notably, the reduced EPS contents were primarily caused by the decrease in the PN content under HM stress, while the PS content remained relatively stable, implying that PN might contribute to maintaining structural stability (Shi et al., 2017). Moreover, the TB-PN content was substantially higher compared to that of SMP-PN and LB-PN, which accounted for a considerable proportion of the total PN (Figure 4B), potentially playing a crucial role in granule stability and HM adsorption. However, prolonged operation led to a slight decrease in the TB-PN content, which was mainly due to the transformation of TB-PN to SMP-PN and LB-PN. The overall PS content showed an increasing trend followed by a decrease (Figure 4C), particularly in SMP-PS, which correlated with the microbial activity in the AGS interior, with a higher activity resulting in a higher secretion of SMP-PS. Under prolonged HM stress, the LB-PS and TB-PS contents also exhibited a minor decrease.

#### 3.3.2 Changes in c-di-GMP content and correlation analysis with EPS, VSS, COD removal

**Figure 5:**
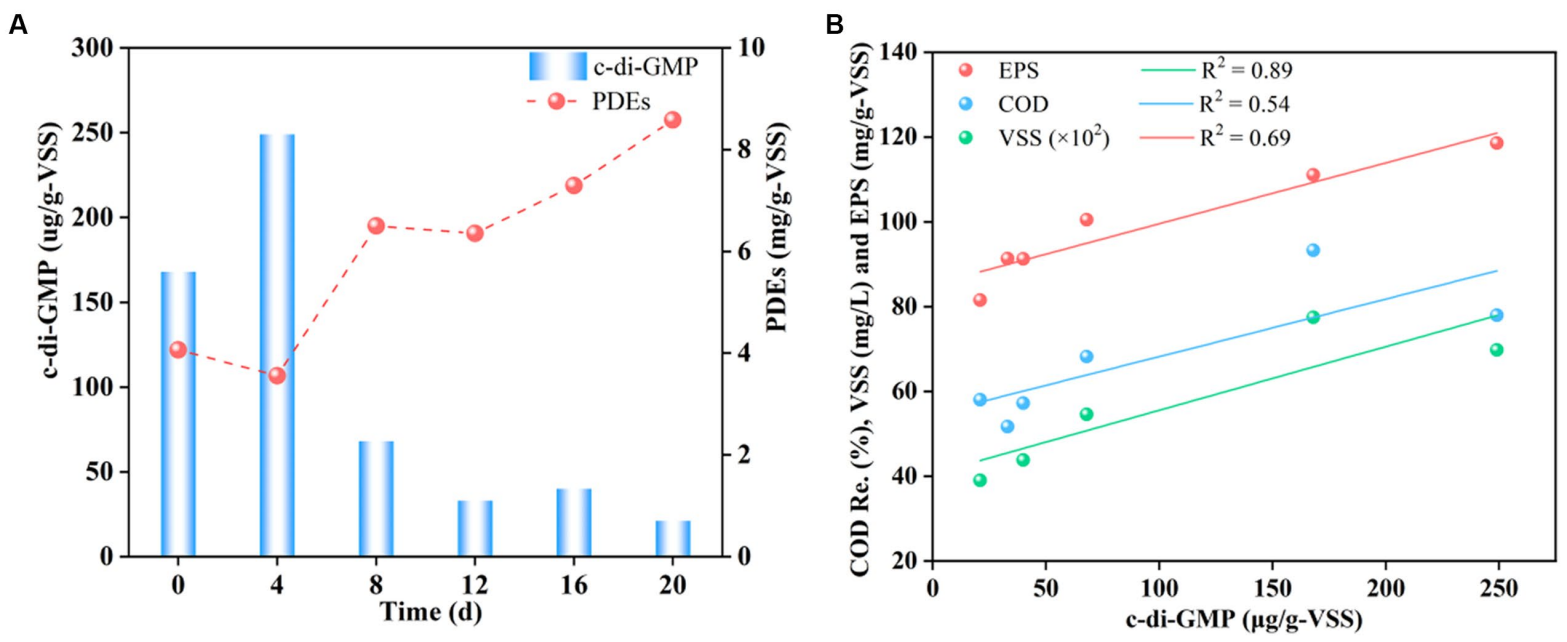
Changes in the c-di-GMP content **(A)** and correlation analysis with EPS, VSS, COD removal **(B)**.

Granule stability was likely related to the EPS content, which in turn was regulated by c-di-GMP levels (Shi et al., 2017; Wu D. et al., 2023; Wu X. et al., 2023). Figure 5 shows the contents of intracellular c-di-GMP and the correlation between EPS, VSS, COD removal, and c-di-GMP under complex HM stress. As shown in Figure 5A, the content of c-di-GMP increased from 168 to 249 ug/g-VSS on the 4th day after HM addition, decreased sharply to 68 ug/g-VSS on the 8th day, and finally decreased gradually to a minimum level of 21 μg/g-VSS. These results indicated that the addition of HM initially triggered an increase in c-di-GMP levels but then resulted in a decrease, with an overall negative correlation with c-di-GMP levels. The initial rise in c-di-GMP levels was attributed to microbial defense mechanisms against HM-containing wastewater, stimulating the secretion of various signaling molecules due to enhanced microbial activity. For example, Ca(II) addition promoted c-di-GMP secretion, while Mn(II) aided in the synthesis of PDEs to hydrolyze c-di-GMP, leading to reduced levels, as PDEs function in the hydrolysis of c-di-GMP (Wang et al., 2017b). Later, a substantial amount of HM ions diffused into the cells, activating the synthesis of PDEs. This increase, from the initial 4.0 to 8.5 mg/g-VSS, resulted in the degradation of c-di-GMP into smaller molecules to render it non-functional as a messenger (Wan et al., 2013), leading to a sharp decline in c-di-GMP levels. It is well-known that the decrease in c-di-GMP slows down the synthesis of EPS, explaining the reduction in the EPS content (Figure 4).

Further revealed correlations between EPS, SS, COD removal, and c-di-GMP using kinetic fitting, with correlation coefficients of 0.89, 0.69, and 0.54, respectively (Figure 5B). The results confirmed the strongest correlation between the EPS content and c-di-GMP levels, while the weakest association was found for COD removal. The high correlation may be explained by the fact that c-di-GMP primarily functions as a secondary messenger and as a microbial signaling molecule that facilitates the transmission of messenger information, so that it can directly act on and cause a response of EPS-secreting microorganisms. However, pollutant removal is influenced by various factors such as pollutant concentrations, pH, dissolved oxygen, or granule structure. As c-di-GMP affected the pollutant removal by impacting the EPS content and thus granule morphology, the correlation between c-di-GMP and COD removal was likely the lowest. Collectively, HM addition diminished c-di-GMP levels, and the EPS content was directly related to c-di-GMP levels. High levels of c-di-GMP positively affected the synthesis of EPS and maintained the structural stability of granules, while low levels of c-di-GMP caused granule disintegration through a reduction in the EPS content.

### 3.4 Mechanisms analysis underlying the removal of complex HM

#### 3.4.1 Ion exchange

**Figure 6:**
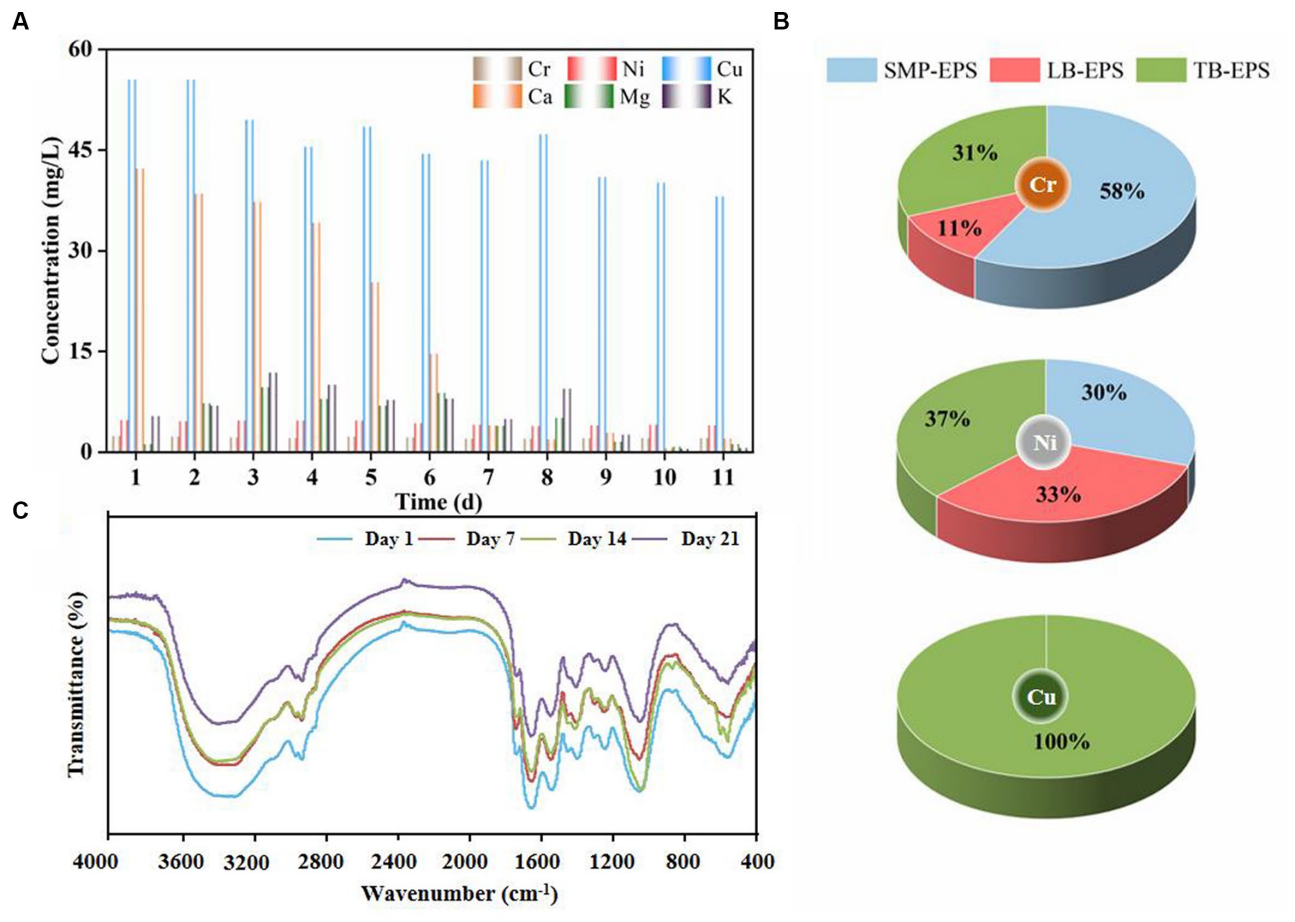
Concentration of heavy and light metal ions in solution **(A)** and proportional distribution of Cu(II), Ni(II), and Cr(III) in EPS fractions **(B)** and FTIR spectra **(C)**.

Figure 6A presents the evolving concentrations of heavy and light metal ions in solution. The content of light metal ions such as Ca(II), Mg(II), and K(I) gradually increased with the removal of Cu(II), Ni(II), and Cr(III) at the beginning of the experiment, indicating that absorption of complex HM displaced light metal ions by means of ion exchange. Specifically, the quantity of released Ca(II) ions was significantly higher than that of Mg(II) and K(I) ions and in the order Ca(II) > Mg(II) > K(I). This is consistent with previous findings that a substantial portion of Ca(II) ions originated from ion exchange with HM such as Cu(II) and Ni(II) (Liu and Xu, 2007; Xu and Liu, 2008). Indeed, Ca(II) and Mg(II) ions served as stimulants for the microbial secretion of PN and PS, which corresponds to the initial rise in the EPS content depicted in Figure 4, and had a positive effect on maintaining granular structural stability (Kończak et al., 2014). As the experiment proceeded, the release of light metals gradually decreased, probably due to saturation of displacement sites by HM, resulting in a decline in the ion exchange rate. However, HM were still continuously removed, implying that ion exchange was not the only removal mechanism. Cu(II) and Cr(III) potentially engaged in chemical reactions with substances in aerobic granules to generate precipitates such as Cu_2_(OH)_3_Cl, Cr(OH)_3_, and CrxMg_1.5_(1-x)(OH)_3_ (Xu and Liu, 2008; Liang et al., 2021), which may explain the color variation in the granules and the formation of scale-like entities (Supplementary Figure S2).

#### 3.4.2 Adsorption and transfer of HM in EPS fractions

To reveal the adsorption and transformation mechanisms of Cu(II), Ni(II), and Cr(III) per EPS fraction in detail, the content and proportion of HM in individual EPS fractions was examined (Table 1 and Figure 6B). The results indicated that EPS adsorption played a pivotal role in HM removal. Cu(II), Ni(II), and Cr(III) were initially adsorbed in SMP-EPS and gradually diffused into LB-EPS (except for Cu(II)) and TB-EPS since HM ions are more readily available in SMP-EPS than in bound EPS (Li G. et al., 2020; Li N. et al., 2020). Particularly, approximately 60% of Cr(III) was removed by adsorption on SMP-EPS (Figure 6B), highlighting its critical role in Cr(III) adsorption, while LB-EPS only adsorbed 11% of Cr(III). Cu(II) was initially adsorbed by SMP-EPS and subsequently diffused entirely to TB-EPS, without detection of Cu(II) in LB-EPS, highlighting TB-EPS as the crucial layer for Cu(II) removal. Ni(II) was adsorbed in equal proportions on SMP-EPS, LB-EPS, and TB-EPS. Additionally, based on EPS adsorption capacities and adsorption sequences for Cu(II), Ni(II), and Cr(III), the order of their affinities for EPS was Cu(II) > Ni(II) > Cr(III), whereas that for the total amount adsorbed on EPS was Cu(II) > Cr(III) > Ni(II). Notably, the adsorption concentration of Cu(II) exceeded 200 mg/L. In summary, SMP-EPS and TB-EPS were identified as the pivotal layers for Cr(III) and Cu(II) adsorption, respectively, whereas Ni(II) was uniformly adsorbed across SMP-EPS, TB-EPS, and LB-EPS.

**Table 1:** Contents of Cu(II), Ni(II), and Cr(III) in EPS fractions.

Time (d)	Cr(III) (mg/L)			Ni(II) (mg/L)			Cu(II) (mg/L)		
	SMP	LB	TB	SMP	LB	TB	SMP	LB	TB
0	0	0	0	0.3	0	0	18.6	0	0
4	0.4	0	0	0.8	0.9	0	29.7	0	28.6
8	1.2	0.6	1.2	1.1	0.9	0.3	22.6	0	21
12	2.1	0.5	1.3	0.7	0.6	0.8	12	0	117
16	3.4	0.7	1.2	0.7	0.8	1.1	4	0	193.8
20	2.6	0.5	1.4	0.8	0.9	1.0	0	0	215.4

#### 3.4.3 Bonding to functional groups

EPS possess high binding capacity for HM due to their abundant functional groups, such as carboxyl, amino, and hydroxyl groups, and have been proven pivotal in the adsorption of Cu(II) and Ni(II) (Sheng et al., 2010, 2013; Li et al., 2017). Figure 6C shows the functional groups identified in the FTIR spectra of AGS in the range of 400–4,000 cm^−1^ 1, 7, 14, and 21 d after HM addition. The stretching absorption band at 3,425 cm^−1^ was observed to be broadened, which likely attributed to the stretching vibrations of -OH and N-H in PS. Distinct fluctuations at 1,734 cm^−1^ implied possible interactions between HM and C=O in carboxyl groups. Additionally, the shift of the C-O stretching vibration band in the carboxyl group from 1,400 cm^−1^ to 1,380 cm^−1^ may be attributed to the interactions between Cr(III), Cu(II), and Ni(II) and the bending modes of C-O-H originating from alcohol groups (Chen et al., 2002). The amide I region (1,700–1,600 cm^−1^) has been widely utilized to quantify the secondary structural composition of PN and peptides (Lin et al., 2018). Remarkably, the spectral band at 1,640 cm^−1^ remained relatively unchanged, suggesting that HM addition did not significantly disrupt the secondary structure of PN. The intensive absorption bands at 1,550 cm^−1^, representing the deformation vibrations of N-H and C-N, indicated the formation of more coordinated metal ions and protein complexes with functional groups such as -OH and -COOH in PN. The stretching of C-OH at 1,047 cm^−1^ shifted to higher wavenumbers after addition of HM, signifying the relevance of amino and hydroxyl groups during the adsorption process. In conclusion, the major functional groups exhibited no significant changes after the addition of HM, underscoring the high stability of AGS in HM removal.

#### 3.4.4 Inner-sphere adsorption of HM

Apart from ion exchange and adsorption on EPS fractions, the observation of scale-like entities in the granule interiors (Figure 1). It was examined the contribution of HM in the granule interiors in detail to elucidate their fate within these granules (Figure 2). A substantial increase in the internal Cu(II) content from 0 to 7% was noted (Figures 2A–D), while lower proportions of Ni(II) (1%) and Cr(III) (1%) were detected in the second and third week, respectively (Figures 2C,D). This outcome indicated that Cu(II), Ni(II), and Cr(III) were all capable of penetrating into the granule interior to undergo inner-sphere adsorption, with the penetration rate being in the order Cu(II) > Ni(II) > Cr(III). The ability of HM to penetrate into the granule interiors for adsorption is mainly due to the abundant pore channels within the granules (Ahn and Hong, 2015). However, the internal content of Ni(II) and Cr(III) was relatively lower than those of Cu(II), possibly owing to their low influent concentration, most of which was removed by EPS adsorption outside the cell. In addition, variations in Ca(II), Mg(II), K(I), and Na(I) ion contents are important as well. The concentration of Ca(II) ions decreased gradually from 21 to 13% (Figure 2), mainly due to ion exchange with HM. In toxic wastewater treatment, Ca(II) ions can serve as bridging connections for hydroxyl groups in the cell membrane protein and phospholipids in the membrane, forming a protective layer that safeguards the cell membrane. Mg(II) ions, acting as enzymatic promoters, play a crucial role in sustaining microbial activity in sludge and enhancing resistance to HM. Generally, cell membranes regulate Na(I) and K(I) ions to maintain osmotic pressure and membrane potential balance inside and outside the cells. Under HM stress, Na(I) and K(I) ion contents remained relatively stable (Figure 2), indicating the normal functioning of microbial cell membranes in AGS, which holds paramount significance in ensuring the stable operation of the AGS process.

### 3.5 Removal mechanism of complex HM by AGS and potential for recycling applications

**Figure 7:**
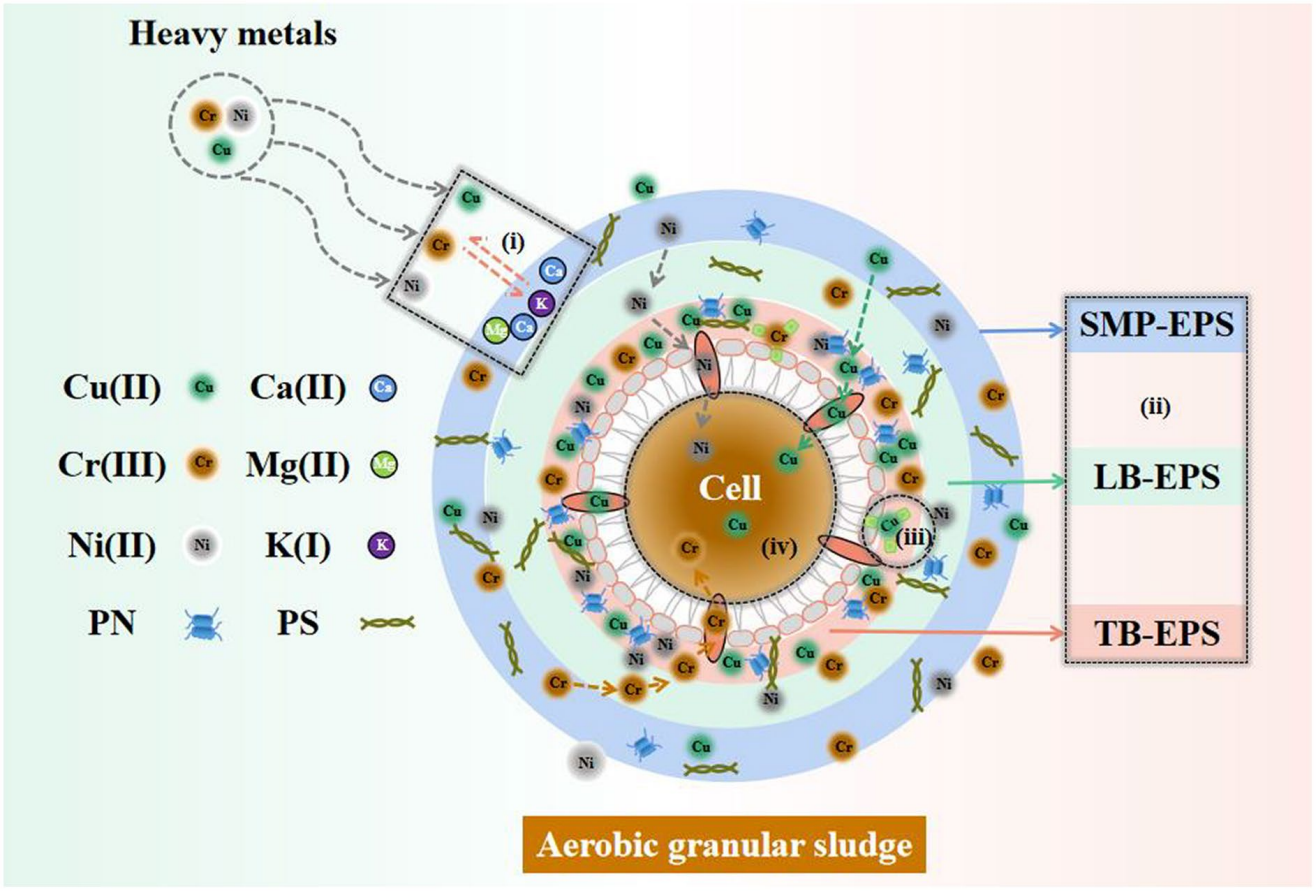
Potential removal mechanism of complex HM by AGS. (i) Ion exchange, (ii) Three-layer EPS adsorption, (iii) Slight surface precipitation, (iv) Inner-sphere adsorption.

The comprehensive removal mechanism of complex HM by AGS is illustrated in Figure 7. Cu(II), Ni(II), and Cr(III) initially engage in extracellular ion exchange with the light metals Ca(II), Mg(II), and K(I), after which all HM are adsorbed on SMP-EPS. Then, Ni(II) and Cr(III) progressively diffuse from SMP-EPS to LB-EPS and TB-EPS, while Cu(II) directly diffuses from SMP-EPS to TB-EPS. A majority of HM ions were removed through adsorption on the three EPS fractions, while the remaining HM ions entered into the granule interior through cellular pore channels to undergo inner-sphere adsorption. This mechanism removed Cu(II), Ni(II), and Cr(III) efficiently, with Ni(II) being removed almost completely.

HM removed on EPS adsorption can be further utilized for metal recycling through adsorption–desorption cycles. Research has demonstrated remarkable recovery rates of Cu(II) and Pb(II) ions adsorbed on EPS, with proportions as high as 86 and 90%, respectively, underscoring the remarkable metal recovery potential of EPS adsorbents (Ajao et al., 2020). However, isolation of specific metals from complex HM streams is particularly challenging because identification of components with selective metal ion binding ability and discovery of extracellular PN endowed with metal-binding characteristics is difficult (Hasani Zadeh et al., 2023). For example, a significant portion of Cu(II) and Cr(III) was adsorbed on TB-EPS and SMP-EPS in this study, necessitating a focused approach to separately recover Cu(II) and Cr(III) from TB-EPS and SMP-EPS for further re-utilization. Therefore, optimizing process conditions is of paramount importance for the selective adsorption of HM on EPS. Furthermore, the binding affinity of HM ions to functional groups must be considered. Notably, significant competition exists among HM ions for EPS binding sites (Pagliaccia et al., 2022). Genetic engineering can help identify functional EPS-PN and characterize their metal binding affinity to further enhance metal recovery efficiency and convenience. Ultimately, feasibility assessments for the application of EPS with distinctive HM binding affinity in bio-recovery can be advanced through extensive genomics or bioengineering research.

## 4 Conclusion

This study delved into the relevance of the structure, performance, and mechanisms of AGS in the removal of complex HM from wastewater, affirming its excellent applicability. The findings revealed that AGS efficiently removed Cu(II), Ni(II), and Cr(III) through the orchestrated mechanisms of ion exchange, three-layer EPS adsorption, and inner-sphere adsorption, in which Ni(II) was almost completely (>99%) removed. Further revelations indicated the pivotal role of three-layer EPS adsorption in the removal of HM, followed by ion exchange and inner-sphere adsorption. The crucial fractions for adsorbing Cr(III) and Cu(II) were determined to be SMP-EPS and TB-EPS, while Ni(II) was uniformly adsorbed on SMP-EPS, TB-EPS, and LB-EPS. Additionally, the affinity of complex HM for EPS was in the order Cu(II) > Ni(II) > Cr(III). Finally, it could demonstrate that the EPS content was directly regulated by c-di-GMP, thus altering the structural composition of granules. This study unveils the favorable adaptability of AGS to complex HM-containing wastewater, elucidates the underlying removal mechanisms, and provides valuable insights for practical engineering applications of bio-remediation processes for HM.

## Data availability statement

The original contributions presented in the study are included in the article/Supplementary material, further inquiries can be directed to the corresponding author.

## Author contributions

SW: Writing – review & editing, Funding acquisition, Resources, Supervision. CL: Writing – review & editing, Funding acquisition, Visualization. YS: Writing – original draft, Data curation, Methodology, Software, Writing – review & editing. YL: Writing – review & editing, Supervision, Validation. FH: Writing – review & editing, Conceptualization, Visualization. JL: Writing – review & editing, Funding acquisition, Resources, Visualization.
