# Supplementary material for: Aerobic granular sludge for complex heavy metal-containing wastewater treatment: characterization, performance, and mechanisms analysis
Source: Front Microbiol. 2024 Jan 31;15:1356386. doi: 10.3389/fmicb.2024.1356386 (PMC10864496; doi:10.3389/fmicb.2024.1356386)
Supplement: Supplementary file 1 [file Data_Sheet_1.docx]

***Supplementary Material***

**Aerobic granular sludge for complex heavy metal-containing wastewater treatment: characterization, performance, and mechanisms analysis**

**Chong Liu ^1^, Yao Shen ^2^, Yuguang Li ^1^, Fengguang Huang ^1^, Shuo Wang ^23*^, Ji Li ^23^**

^1^101 Institute of the Ministry of Civil Affairs, Key Laboratory of Embalming Methodology and Cosmetology of Cadavers of the Ministry of Civil Affairs, Beijing 100070, China

^2^Jiangsu Key Laboratory of Anaerobic Biotechnology, School of Environment and Ecology, Jiangnan University, Wuxi 214122, China

^3^Jiangsu College of Water Treatment Technology and Material Collaborative Innovation Center, Suzhou 215009, China

*** Correspondence:**

*Corresponding author: Shuo WANG

E-mail: shuowang@jiangnan.edu.cn

**1 Supplementary Figures**


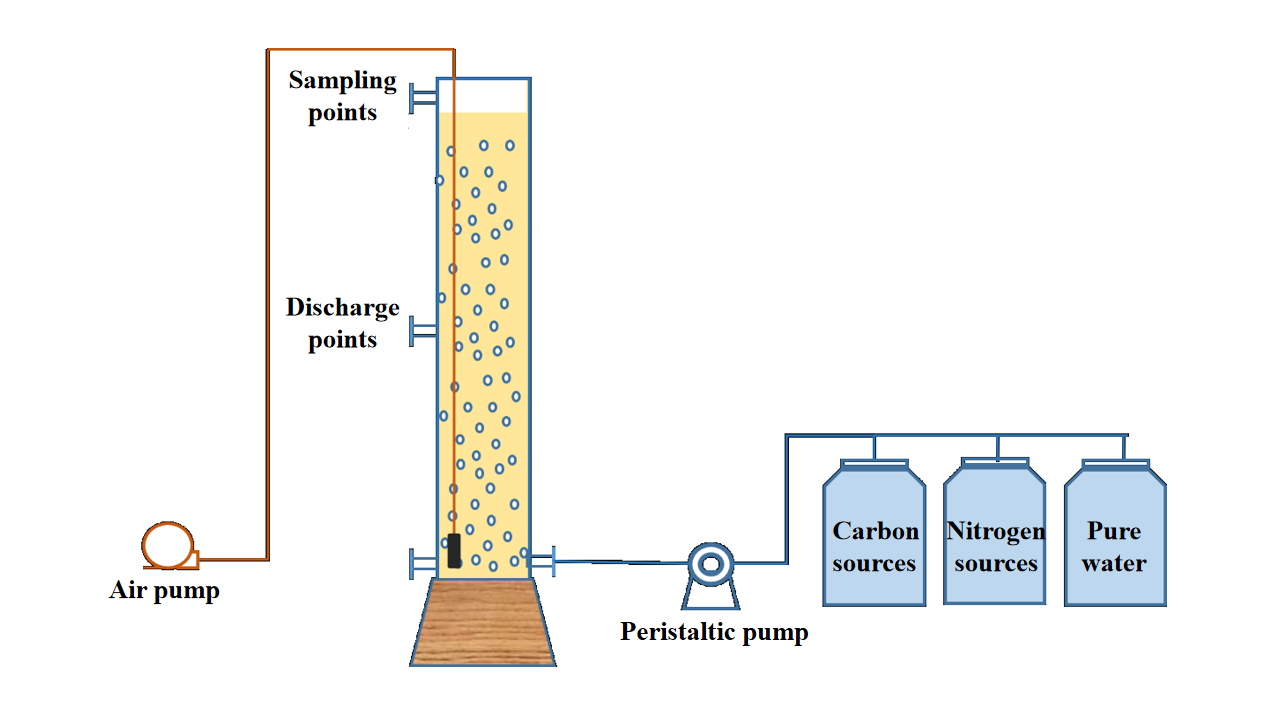


**Supplementary Figure 1.** Schematic diagram of the aerobic granular sludge sequencing batch reactor.


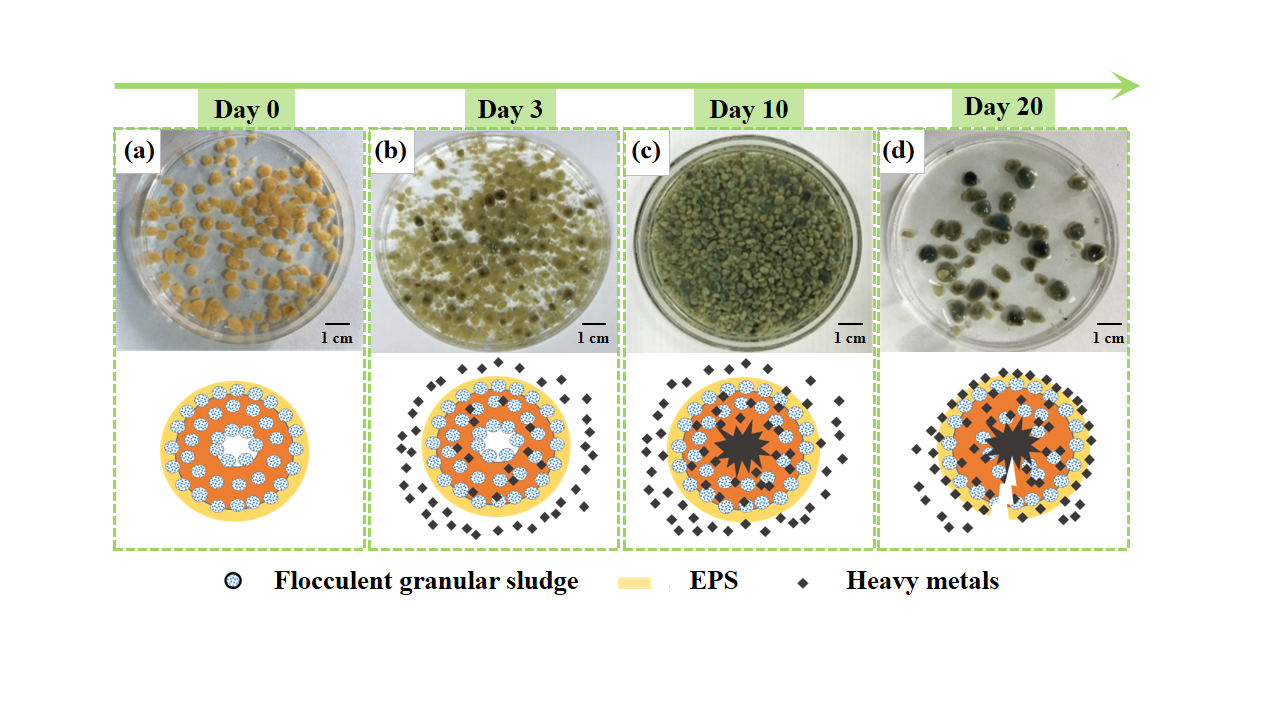


**Supplementary Figure S2.** Variations in morphology of AGS after HM addition.


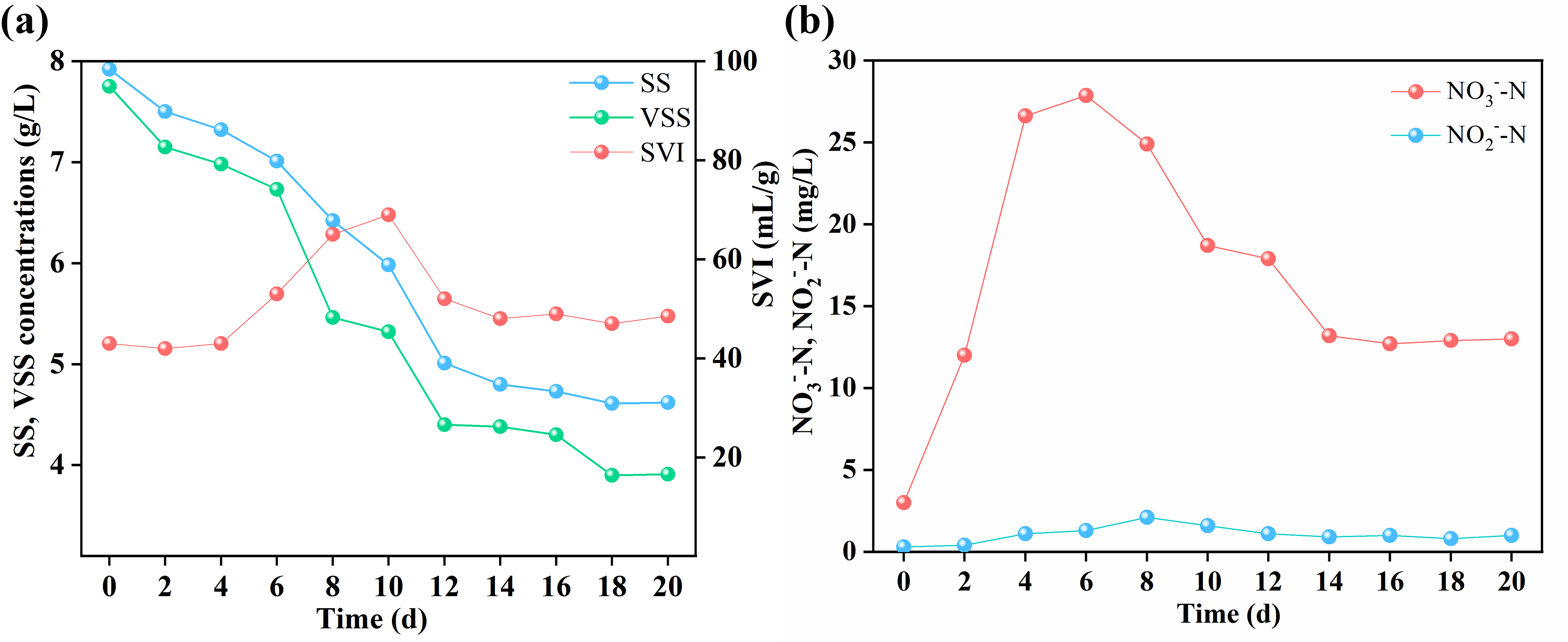


**Supplementary Figure S3.** Variations in SS, VSS, SVI, NO_3_^-^-N, and NO_2_^-^-N contents.
